# The Impact of Certificate of Need Laws on Home Health Care Access for Medicare Beneficiaries With Dementia

**DOI:** 10.1093/geroni/igaf122.282

**Published:** 2025-12-31

**Authors:** Jacy Weems, Xiao (Joyce) Wang, Katherine Ornstein, Anna Beeber, Jamie Smith, Momotazur Rahman

**Affiliations:** Brown University School of Public Health, Providence, Rhode Island, United States; Brown University, Providence, Rhode Island, United States; Johns Hopkins University, Baltimore, Maryland, United States; Johns Hopkins University, Baltimore, Maryland, United States; Widener University, Philadelphia, Pennsylvania, United States; Brown University, Providence, Rhode Island, United States

## Abstract

Certificate of Need (CON) laws aim to control state healthcare spending by regulating the establishment of healthcare facilities based on demonstrated need. While CON laws have been largely ineffective in regulating hospital and nursing homes, emerging evidence suggests they may reduce home health care use. Currently, 14 states maintain CON regulations for home health services. Persons with Alzheimer’s disease and related dementias (ADRD) increasingly rely on Medicare skilled home health care, especially through community-initiated home health services, to age in place. In this study, we examined whether CON laws reduce home health use among persons with ADRD. We analyzed data from 818,878 Medicare fee-for-service beneficiaries newly diagnosed with ADRD in 2016, tracking their healthcare use over 48 months before and after diagnosis. Using an individual fixed-effects difference-in-differences model, we compared changes in utilization between states with and without home health CON laws. Before ADRD diagnosis, home health use per person per month was 5.9%. After diagnosis, utilization increased by 7.7 percentage points in states without home health CON and by 6.9 percentage points in CON states, suggesting that CON laws lead to approximately 10% lower home health use among individuals with ADRD. We did not observe a significant impact of CON laws on hospital and nursing home utilization. Given CON laws are associated with comparatively less home health utilization among individuals with ADRD, further research is needed to understand how this affects patient outcomes.

